# Monitoring of *Bacillus* spore-forming dynamics through flow cytometry

**DOI:** 10.3389/fmicb.2024.1450913

**Published:** 2024-10-29

**Authors:** Zhili Chen, Yuanyuan Lu, Jiazhen Cui, Yuzhong Feng, Haolong Dong, Xuan Huang, Chen Zhu, Xianghua Xiong, Huipeng Chen, Qingyang Wang, Gang Liu

**Affiliations:** ^1^Academy of Military Medical Sciences, Beijing, China; ^2^Institute of Physical Science and Information Technology, Anhui University, Hefei, China

**Keywords:** flow cytometry, *Bacillus thuringiensis*, spore forming rate, plate counting, microbial pesticide

## Abstract

The plate counting method is a traditional and widely accepted technique for live cell counting, often employed for *Bacillus* enumeration and spore forming rate calculations. However, this method requires at least 12 h to generate results, making it unsuitable for real-time monitoring of bacterial growth status and spore transformation rate. *Bacillus thuringiensis* crystals, produced during sporulation, are widely used as microbial pesticides, with high demand for industrial scale production. Variations in cultivation conditions and harvest timing during large-scale pore production of *Bacillus thuringiensis* significantly affect spore forming rate, impacting crystallization yield. Nevertheless, there is a lack of real-time monitoring methods for spore conversion rate. Flow cytometry (FCM), a well-established technique for single-cell analysis in eukaryotic cells, has been successfully applied in bacterial detection in environmental and food samples. In this study, we introduced a rapid flow cytometry-based method for determining spore forming rate of *Bacillus thuringiensis*, with two nucleic acid dyes, SYTO24 and LDS751. The method enables dynamic monitoring of spore, vegetative cell, and viable but non-culturable/dead cell proportions during the whole cultivation process, and spore forming rate could be gained within 30 min. Data of spore forming rate by FCM method is consistent with that by plate counting method, offering a faster and more efficient approach for assessing sporulation status in industrial *Bacillus thuringiensis* microbial pesticide production.

## 1 Introduction

Pests are a primary cause of agricultural losses during cultivation, storage and distribution of crops (Vivekanandhan et al., 2023). Conventional ways to control pest mainly relies on insecticide spraying, which not only leads to environmental pollution but also poses risks to human health (Ayilara et al., 2023; Fiaboe et al., 2023). Microbial pesticides, due to their eco-friendliness and environmental sustainability, have garnered great attention among researchers (Bel et al., 2020). *Bacillus thuringiensis* (*Bt*) products have emerged as highly effective microbial pesticides due to their diverse species resources, low drug resistance, and minimal environmental toxicity (Sauka et al., 2023). *Bt* is a Gram-positive and spore-forming bacterium that is pathogenic to insects. It exhibits potent pesticidal activity and high toxicity with low cost (Bora et al., 2023; Nair et al., 2021). Under specific nutrient-deficient growth conditions, *Bt* can form circular or elliptical dormant spores within its cells (Zhang et al., 2017). Different subspecies of Bt produce varied structures of insecticidal protein crystals known as parasporal crystals during spore formation (Tetreau et al., 2021). As a result, the endophytic spores and crystals of *Bt* are extensively utilized in insecticidal research and represent the most commonly used microbial pesticides globally, meeting substantial industrial demand (Biermann and Beutel, 2023; Bressuire-Isoard et al., 2018; Pérez-García et al., 2011).

In modern industry, determination of sporulation is typically assessed through empirical observation. Sporulation efficiency is defined as the percentage of endospore cells generated within cultured cells. Enhancing the spore forming rate and minimizing material wastage are crucial for industrial demands in the microbial product market (Biermann et al., 2023). Factors influencing spore forming rate include the composition of the culture medium, cultivation conditions, and biological processes (Biermann and Beutel, 2023). Industrial expertise is developed by iteratively optimizing parameters in small-scale laboratory settings before scaling up to industrial production. Spore forming rate is commonly evaluated in optimization experiments using the plating method, which involves quantifying the number of colony-forming units from bacterial liquid on solid plates pre- and post-heat shock treatment (Paidhungat and Setlow, 2000). However, the plating method has limitations such as operator variability, extended processing time, and limited applicability to certain bacteria like viable but non-culturable (VBNC) strains (Ambriz-Aviña et al., 2014; Khan et al., 2010)

Flow cytometry (FCM) is a high-throughput technique used for rapid detection and analysis of single cell, as well as purification of cell subpopulations based on fluorescence intensity and light scattering properties. Flow cytometry is widely utilized in immunology, molecular biology, microbiology, virology, cancer biology, and infectious disease monitoring (McKinnon, 2018). Recently, its applications have expanded to bacterial counting, vitality assessment, water quality testing (De Roy et al., 2012) fermentation manufacturing (Bühligen et al., 2014), dairy production (Sheehan et al., 2005), probiotic production (Davis, 2014), and quantification and isolation of *Bacillus* spores (Karava et al., 2019), Genovese et al. (2021) conducted flow cytometry to determine *Bacillus* spore forming rate, successfully distinguishing spores from vegetative cells and dead cells. However, this method is established by measuring a mixture comprised of known proportion of spore and vegetative cell and does not show real-time monitoring of the dynamic of spore formation process. In this study, we developed a rapid and dynamic monitoring approach to track spore production in *Bt* during spore formation by using flow cytometry. We systematically evaluated the accuracy of this approach by comparison with plate counting and spore staining methods. The entire process, from sampling to obtaining results, was completed within 30 min, significantly reducing the time consumption compared to conventional plate counting method.

## 2 Materials and methods

### 2.1 Strains and reagents

The *Bacillus thuringiensis* subsp. *thuringiensis* (BT03) utilized in this research was acquired from the China Center for the Preservation and Management of Common Microbial Strains. The LB Broth culture medium was sourced from Coolber Corporation in China, and the SYTO24 and LDS751 dyes were purchased from ThermoFisher.

### 2.2 Cultivation of *Bacillus thuringiensis*

Frozen stocks of *Bt* were thawed at room temperature and cultured on LB agar plates at 30°C. Bacteria were transferred into 4 ml of LB broth (consisting of 10 g/L tryptone, 5 g/L yeast extract, and 10 g/L sodium chloride, with a pH of 7.0), and were incubated at 30°C with a rotation speed of 200 rpm for about 12 h. Subsequently, the bacterial fluid was centrifuged at 12,000 rpm for 5 min, the supernatant was discarded, and the precipitate was resuspended in 2 mL of LB. The optical density (O.D.600) of the resuspension was determined at a wavelength of 600 nm. An inoculum with an O.D.600 value of 0.6 at a 1/100 ratio was added into a 200 mL non-resistant LB shake flask, and incubated at 30°C and 200 rpm for 5 days. Each set of experiments was replicated in triplicate using three shake flasks.

### 2.3 Heat-inactivation of *Bt* samples

Samples were collected at 24, 48, 60, 72, and 96 h after the initiation of *Bt* cultivation. Samples from each flask were divided into two EP tubes with one being subjected to 65°C heat treatment for 35 min (referred to as heat-inactivated) while another being kept in room temperature for same time (referred to as normal).

### 2.4 Plate counting method

Vigorously vortex samples in EP tubes for 20 s. Diluted the samples by a factor of 10 using a continuous dilution method, reducing the concentration from 10^–1^ to 10^–8^. Between each dilution, vigorously vortex samples for 20 s. 100 μL of bacterial suspension from each dilution it was plated on non-resistant LB agar plates and incubated at 30°C.12 to 16 h later, visible colonies on plates with a appropriate count ranging from 30 to 300 were counted and the colony-forming units (CFU) per milliliter (CFU/mL) were calculated. Spore forming rate was determined as the CFU of inactivated samples divided by the CFU of room temperature samples, represented as a percentage form.

### 2.5 Spore staining and microscopic examination

Transferred 40 μL of the room temperature group’s original bacterial suspension to an EP tube and centrifuged at 12,000 rpm for 5 min to enrich bacterial cells at the tube’s bottom. Discarded the supernatant and resuspend the cells in 100 μL of non-antibiotic LB culture medium. Spread 20 μL of the resuspended bacterial fluid evenly on a clean glass slide, heat-fix for 2 min, then stained using a ZOPOMED automatic Gram staining machine. Performed primary staining with malachite green dye for 15 min, followed by secondary staining with safranin dye for 10 s at 95°C. Rinsed the slide with running water, dried it, and observed under an oil microscope. Bacterial cells appeared purple-red, while spores appeared blue-green.

### 2.6 Sample preparation for FCM

Two fluorescent dyes, SYTO24 and LDS751, were utilized for distinguishing spores, vegetative cells and VBNC/dead cells (Genovese et al., 2021). The bacterial liquid sample for flow cytometry was prepared at a volume of 400 μL. Initially, the samples from the normal group and inactivation group were shaken for 20 s, followed by the extraction of 100 μL, which was then mixed with 900 μL of non-antibiotic LB culture medium for dilution. Subsequently, 40 μL of the diluted bacterial suspension was transferred to a flow cytometry tube containing 280 μL of sterile water. 40 μL of SYTO24 dye at a concentration of 100 nmol/L was added to achieve a total volume of 360 μL in the flow cytometer. After shaking for 10 s, the mixture was incubated at 37°C in darkness for 10 min. Finally, 40 μL of LDS751 dye at a concentration of 200 ng/mL was added, followed by another round of shaking for 10 s and an additional incubation at 37°C in darkness for 5 min before use. Before commencing flow analysis, make sure to shake and mix the sample thoroughly. The SYTO24 and LDS751 single staining groups are adjusted with physiological saline to reach a total volume of 400 μL.

### 2.7 Flow cytometry

Cytometric analyses were done using a BD FACSCelesta with a 488 nm argon laser. The SYTO24 dye has an excitation peak at 490 nm and an emission peak at 515 nm. Excitation is achieved using a blue laser at 488 nm, with emission light signals collected through a 530/30 filter, known as the FITC channel. The LDS751 dye, with excitation peak at 543 nm and emission peak at 712 nm, is excited by a blue laser with 53% efficiency. Emission signals are captured using a 695/40 filter, designated as the 7-AAD channel. Machine parameters remain consistent for all time point samples, with FSC set at 753, SSC at 400, FITC at 360, and 7-AAD at 600. The compensation setting for 7-AAD-FITC is 20. Define the gate enclosing the primary cell cluster and collect 10,000 cells within the gate.

### 2.8 Statistical analysis

The GraphPad Prism 8.0 (San Diego, CA) software package was used for statistical analysis in this study. The Student’s *t*-test was used to compare the difference between two groups. *P* < 0.05 was considered as a significant difference.

## 3 Results

### 3.1 Monitoring sporulation efficiency through plate counting method

**FIGURE 1:**
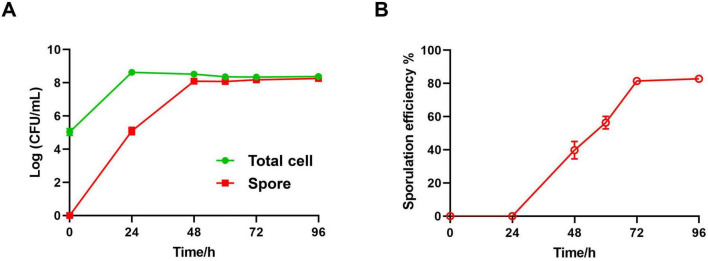
Evaluation of sporulation efficiency by plating method. **(A)** The concentration of total cells (green line) and spores (red line) in LB liquid medium are measured by counting the viable colony forming unit on LB agar plate. Data are shown in a Log CFU/mL form. **(B)** The sporulation efficiency in total cell is calculated as following: Sporulation efficiency % = Spore counts/Total counts × 100%. Data were summarized from three independent experiments.

Samples of *Bt* in culture were taken every 24 h, and plate counting were performed before and after inactivation. The total viable cell count and spore count were calculated based on dilution factors, from which the spore conversion rate was determined as post-inactivation plate count divided by pre-inactivation plate count. Results showed a rapid increase in total viable cell count within 24 h, peaking at 24 h. Subsequently, there was a slight decrease in total viable cell count until the end of the 96-h culture period, likely due to rapid nutrient depletion, leading to minimal cell death (Figure 1A, green curve). Correspondingly, the spore count reached a plateau at 48 h and gradually increased until 96 h (Figure 1A, red curve). The spore conversion rate, calculated from data at different time points, showed a gradual increase throughout the culture process (Figure 1B), reaching a peak of approximately 82.8% ± 1.0% at 96 h. The trend in spore conversion rate aligns with the growth pattern of *Bacillus* (Gauvry et al., 2021). These findings indicate that *Bacillus thuringiensis* cultured in LB medium contains both spores and vegetative cells at all stages, making it suitable for establishing a flow cytometry dynamic analysis method.

### 3.2 Identification of three cell sub-populations of *Bacillus thuringiensis* by flow cytometry

**FIGURE 2:**
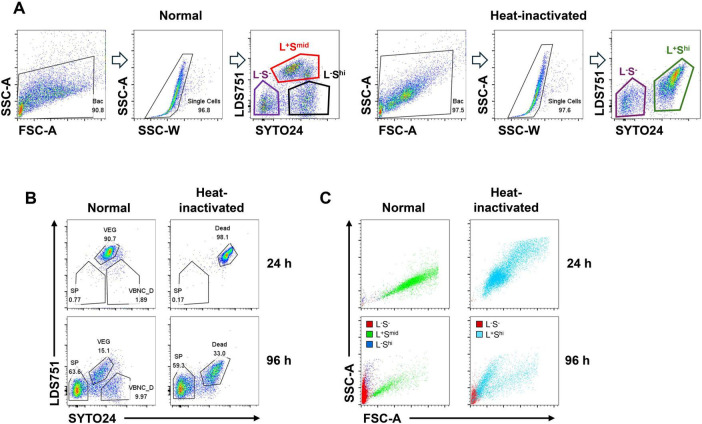
Gating strategy for sub-populations during sporulation by using SYTO24, LDS751, FSC and SSC. **(A,B,C)** Left, Room temperature (Normal) treatment; right, Heat inactivation treatment. **(A)** Firstly, total cells were selected; Secondly, singlets were selected by SSC-W and SSC-A; Finally, cells were separated into three sub-populations by LDS751 and SYTO24. **(B)** Singlets were divided into three distinct sub-populations: LDS751^–^SYTO24^–^ indicates spores (SP), LDS751^+^SYTO24^mid^ indicates vegetive cells (VEG) and LDS751^–^SYTO24^hi^ indicates VBNC and dead cells (VBNC_D). **(C)** Three sub-populations were shown in FSC-A and SSC-A. Red color indicates L^–^S^–^, green color indicates L^+^S^mid^, blue color indicates L^–^S^hi^ and sky blue indicates L^+^S^hi^.

Genovese et al. (2021) successfully distinguished the three cell states of *Bacillus subtilis*: spores, vegetative bodies, and VBNC/dead cells using cell nucleic acid dyes LDS751 and SYTO24 with flow cytometry. On this basis, we used *Bacillus thuringiensis* and its heat-inactivated samples from different culturing stage — the early, vegetative stage (24 h), and the late, spore stage (96 h) — to determine the distribution patterns of different cell sub-populations of *Bacillus thuringiensis* on the LDS751/SYTO24 flow cytometry plot. After gating the main cell cluster and removing the doublets, it was shown that cells from normal treated samples were divided into three sub-populations, LDS751^+^ SYTO24^mid^ (L^+^S^mid^), LDS751^–^ SYTO24^hi^ (L^–^S^hi^), and LDS751^–^ SYTO24^–^ (L^–^S^–^), whereas cells from heat-inactivated samples were divided into two sub-populations, LDS751^–^ SYTO24^–^ (L^–^S^–^) and LDS751^+^ SYTO24^hi^ (L^+^S^hi^) (Figure 2A).

Analysis of flow cytometry data at 24th hour revealed that the proportion of L^+^S^mid^ cell cluster accounted for 90.7% of total cells and was the dominant cell cluster. After inactivation, the proportion of L^+^S^hi^ cell cluster was as high as 98.1%, representing almost all the cells. As reported by literature, the main cell cluster of *Bacillus thuringiensis* after 24 h of culturing is vegetative bodies, indicating that the L^+^S^mid^ cell cluster is a vegetative sub-population (Figure 2B, upper panels). After inactivation, the vegetative bodies became dead cells, with increased permeability to dyes, and the staining shows L^+^S^hi^ (Figure 2B upper panels). By contrast, at 96 h, the majority of cells was characterized as L^–^S^–^, accounting for 63.6% of the total cells. According to literature, spores dominate after 96 h of culturing, which have the minimum permeability to dyes because of their exosporium. It indicated that this cluster of L^–^S^–^ cells are spores. The proportion of L^–^S^–^ cells after inactivation was 59.3%, approximately equal to that of pre-inactivation — a further evidence to prove that the L^–^S^–^ cell cluster represented spore sub-population. L^+^S^mid^ cells decreased from 90.7 to 15.1%, consistent with the pattern of decreased VEG in the late stage of culture, indicating that the L^+^S^mid^ cell cluster represented the sub-population of VEG. According to literature, cell clusters stained as LDS751^–^SYTO24^hi^ are composed of viable but non-culturable cells and dead cells (VBNC and Dead, VBNC_D) (Genovese et al., 2021). In our study, the proportion of L^+^S^hi^ cell clusters after inactivation at 96 h was 33%, which is close to the sum of VEG and VBNC_D before inactivation (Figure 2B lower panels), indicating that the LDS751^–^SYTO24^hi^ subpopulation represented VBNC_D.

Forward scatter (FSC) and side scatter (SSC) parameter analyses on cultured cell sub-populations were conducted to further validate the accuracy of sub-population identification. After transformation into spores, the cell size of *Bacillus subtilis* significantly decreases. The L^–^S^–^ cell sub-population was located in the lower left area of the image, indicating small size and low granularity, which was consistent with the characteristics of spore cells (Figure 2C). By contrast, the sub-population of L^+^S^mid^ cells were mainly distributed in the center of the image, indicating a larger cell size, which was consistent with the characteristics of VEG. The L^–^S^hi^ cell sub-population located between the L^–^S^–^ cells and the L^+^S^mid^ cells, consistent with the characteristics of VBNC_D (Figure 2C). After inactivation, the location of the subpopulations in FSC/SSC diagram matched well with their characteristics (Figure 2C). The analysis of FSC and SSC parameters further confirmed that our cell cluster gating strategy is in line with the characteristics of *Bacillus subtilis*, presenting spores as L^–^S^–^, VEG as L^+^S^mid^, and VBNC_D as L^–^S^hi^.

### 3.3 Utilizing flow cytometry to dynamically analyze spore forming rate

**FIGURE 3:**
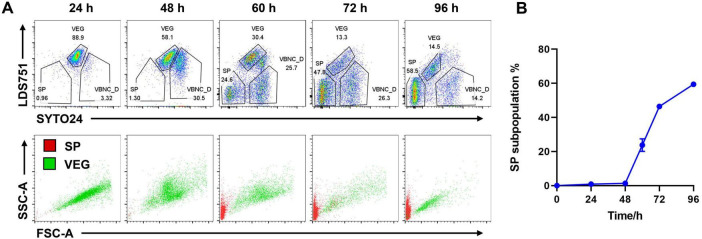
Spore-forming dynamics shown by FCM. **(A)** Samples from different time points during sporulation were analyzed by FCM with SYTO24 and LDS751 staining (upper panels) and FSC and SSC (lower panels). **(B)** Spore proportion dynamics by FCM data. SP subpopulation% was determined by the SP percentage of total singlets in **(A)**. Data were summarized from three independent experiments.

Using the established gating strategy, we sampled *Bt* undergoing continuous cultivation at 24, 48, 60, 72, and 96 h to determine spore conversion rates. Results revealed that within the initial 48 h of cultivation, spore staining was not observed. By the 60 h of cultivation, distinct spore staining appeared, accounting for 31.4% ± 11.9 of the population. The proportion of spore staining continued to increase with cultivation time, reaching 59.4% ± 0.9 by 96 h (Figures 3A, B). Further analysis of FSC and SSC parameters indicated that the spore cluster (red cell cluster) was located in the bottom left corner of the plot, representing very small cell diameters (Figure 3A). The VEG cluster (green cell cluster) gradually moved toward the bottom left of the plot over time, indicating a reduction in cell volume during cultivation (Figure 3A). By making spore forming rate curve (Figure 3B), we successfully established a method to real-time monitor the sporulation status during *Bt* cultivation by utilizing flow cytometry with LDS751 and SYTO24 double staining in combination with FSC and SSC parameters.

### 3.4 Comparing plate counting method with flow cytometry for calculating spore conversion rate

**FIGURE 4:**
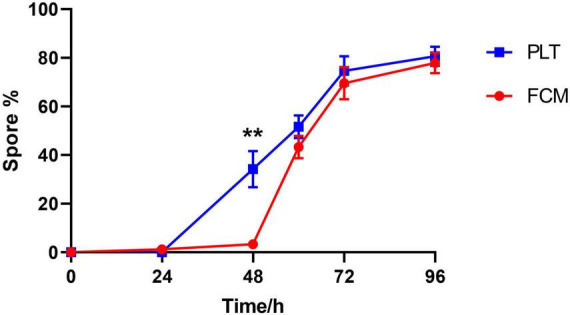
Comparison of spore-forming rate by plating and FCM. The panel showed spore proportion dynamics by FCM and plating methods from three independent experiments, respectively. Spore forming rate in FCM was calculated as following: Spore% = SP%/(SP% + VEG%) × 100%. **P < 0.01.

We further compared the consistency of spore conversion rates of *Bt* calculated with flow cytometry and plate counting method. Plate counting can not detect viable but non-culturable (VBNC) cells and dead cells, which are included in the flow cytometry analysis. Therefore, when comparing spore conversion rates calculated by the two methods, the flow cytometry analysis should exclude the VBNC_D portion and calculate the spore conversion rate as Spore% = SP%/(SP% + VEG%) × 100%. Three independent replicates of flow cytometry and plate counting were conducted, and the experimental data were compared. The dyes LDS751 and SYTO24 showed good stability and reproducibility in distinguishing the three cell populations (Figure 4 and Supplementary Figure 1). The comparison between the two methods for calculating the spore conversion rate further revealed that at the time points of 0, 24, 60, 72, and 96 h, the values obtained for the spore conversion rate were very close. However, at the 48-h time point, the conversion rate measured by plate counting reached 34.2% ± 7.0, whereas flow cytometry showed a conversion rate of only 3.3% ± 0.9. Further analysis revealed that the discrepancy at 48 h was due to early spore formation when the spores were not yet separated from the mother cells. Therefore, after 12 h of cultivation, the “Spore” colonies could be grown, exhibiting a staining pattern of the VEG sub-population under dual staining with LDS751/SYTO24 (El-Khoury et al., 2016; Verplaetse et al., 2015). These results indicate the accuracy, stability, and reproducibility of flow cytometry to determine the spore conversion rates of *Bacillus thuringiensis* in the later stages of cultivation.

### 3.5 A comparative analysis between malachite green/safranin staining and flow cytometry

**FIGURE 5:**
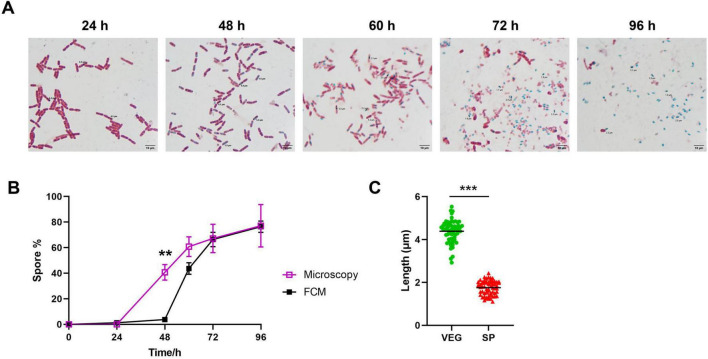
Comparison of endospore staining and FCM. **(A)** Bacterial samples of different time points during sporulation were fixed by steam heat, stained by malachite green and safranin solutions in 95°C and flooded by water. Graphs were taken under a 63 × oil lens by microscopy. **(B)** Determination of spore-forming rate by FCM, and microscopy. Data were collected and presented from three independent experiments. **(C)** Cell length of vegetative cells and spores in **(A)** were measured. A total of fifty cells were measured in each group. **P < 0.01, ***P < 0.001.

Malachite green/malachite green and safranin staining are commonly used for observing spore formation of *Bacillus*. The formed spores are stained with malachite green, appearing blue-green under the microscope (D’Incecco et al., 2015), while the vegetative cells are stained with safranin, appearing purple-red (Liu et al., 2020). Staining of samples at different time points (Figure 5A) reveals that at 24 h, the culture is predominantly rod-shaped bacteria with elongated bodies. By 48 h, around 30% of the bacteria are in the process of spore formation, with spores visible inside the cells as round blue-green structures, although most of them have not yet separated from the mother cells. Spores at this time point remain attached to the cells, which could be the reason why they are able to withstand heat inactivation and form clones in plate counting, but remain in the VEG position in flow cytometry (Figure 3A-48 h, Figure 4-48 h). At 60, 72, and 96 h, the proportion of spores gradually increases, with separation from the vegetative cells (Figure 4); the vegetative cells decrease in size, become more lightly stained, and degrade as the culture ages, consistent with the trend of spore counts increasing shown by flow cytometry (Figures 3A, B). Counting of spores and vegetative cells in stained images under the microscope and calculating the spore conversion rate reveals a curve of conversion rate consistent with that of plate counts; compared to flow cytometry, there is good consistency except at the 48-h time point (Figure 5B). Further measurements of bacterial cell length and spore diameter during the culture process show an average cell length of 4.5 ± 0.3 μm and a spore diameter of 1.9 ± 0.2 μm, indicating significant differences in size (Figure 5C), consistent with the finding in flow cytometry that SP cell clusters are significantly smaller than VEG cell clusters (Figure 3A). These results suggest that the gating strategy for cell clusters in flow cytometry is appropriate and can be used to dynamically monitor the growth status of *Bacillus thuringiensis*.

## 4 Discussion

Currently, the combination of membrane-permeable fluorescent dyes such as SYTO and non-membrane-permeable dye propidium iodide (PI) is commonly employed in flow cytometry for viable cell counting (Sheehan et al., 2005; Shi et al., 2007; Zhao et al., 2017). SYTO dyes stain cells based on membrane permeability (Garcia-Betancur et al., 2012; Wlodkowic and Skommer, 2007), with higher intensity in dead cells compared to live cells and spores. However, due to PI being a non-permeable fluorescent dye, it cannot differentiate between spores, vegetative cells, and cells in a VBNC state in the classification of the sub-populations of *Bacillus thuringiensis*. In our study, SYTO24 and LDS751, substitutes for PI, were used for staining *Bt*. The results revealed that SYTO24 and LDS751 effectively distinguished three sub-populations of *Bt* during spore transformation stages (Figures 2, 3): spores were recognized as L^–^S^–^, vegetative cells as L^+^S^mid^, and VBNC_D as L^–^S^hi^. In a study examining the classification of *Bacillus subtilis* using LDS751 and SYTO24 staining, spore sub-population was identified as LDS751^+^SYTO24 (Genovese et al., 2021). This differs from the L^–^S^–^ sub-population observed in our experiment and may be attributed to variations in *Bacillus* strains and experimental procedures. The study revealed numerous LDS751^–^SYTO24^–^ cells across all experimental groups, indicative of unstained cells. Notably, during the continuous cultivation of *Bt*, the presence of L^–^S^–^ cell clusters was insignificant in the initial 24-h period but became increasingly prominent during the middle and late stages of cultivation (60 h later). This pattern aligns with the bacterial morphology observed under the microscope at different time points (Figure 5A). Further analysis of FSC/SSC parameter combinations for three cell sub-populations revealed distinct patterns (Figures 2B, 3A). The spore sub-population uniformly appears in the lower left corner of the FSC/SSC diagram, indicating smaller cell volume, which conforms to the characteristic of spores. In contrast, the FSC value of VEG decreases gradually over culture time, maintaining an oblique distribution pattern akin to live cells. Notably, VEG cells exhibit significantly higher FSC values than spores, with a clear boundary between the two sub-populations. These findings suggest that the L^–^S^–^cell clusters identified in flow cytometry analysis are spores.

The use of LDS751/SYTO24 for single-cell categorization leads to the development of L^–^S^hi^ cell clusters during the later phases of cultivation, comprising approximately 10 to 30% of the population. A recent investigation suggests that these cell clusters consist of a combination of VBNC and dead cells (VBNC/Dead, VBNC_D) (Genovese et al., 2021). The term VBNC typically denotes a quiescent state of non-spore-forming bacteria in reaction to stressful surroundings, akin to the spore-forming behavior of spore-producing bacteria (van Melis et al., 2011; van Melis et al., 2014). To elucidate the composition and characteristics of the LDS751^–^SYTO24^hi^ cell clusters identified in this research, we inactivated the samples and compared variations in fluorescence spectra pre- and post-inactivation. Following inactivation, theoretically, all cells except spores are expected to be non-viable. Our findings demonstrate that all cells stained as L^+^S^hi^ after 24 hours of inactivation, indicating L^+^S^hi^ as the site of non-viable cells post-inactivation. Calculation reveals that the proportion of L^+^S^hi^ cell clusters post-inactivation approximates the combined sum of VEG and VBNC_D cell clusters pre-inactivation, with no significant alteration in spore cluster proportions before and after inactivation. VBNC_D properties closely resemble those of VEG (Supplementary Figure 1). Further examination of the FSC/SSC distribution of VBNC_D indicates its positioning between spores and vegetations, with a distribution morphology akin to that of VEG. Microscopic examination reveals a notable alteration in bacterial morphology at 60 and 72 h, characterized by a higher prevalence of L^–^S^hi^ cell clusters compared to the initial 24-h cultivation period. The bacteria exhibit a wrinkled, shortened, and thickened appearance at this stage, devoid of spore formation within their bodies. These wrinkled bacteria are hypothesized to be in a viable but non-culturable (VBNC) state. So we speculate that the L^–^S^hi^ sub-population may be mainly composed of *Bt* VBNC and a small portion of dead cells, which could be further confirmed by sorting flow cytometry combined with conventional microscopic techniques. Given the incapacity of VBNC_D to generate quantifiable colonies in plate counting method, adjustments must be made to account for the proportion of VBNC_D cell clusters when determining the spore conversion rate of the sample through flow cytometry analysis.

The Spore conversion rate, calculated as Spore%/(Spore% + Vegetative%) × 100%, demonstrates that flow cytometry and plate counting method exhibit substantial overlap in the advanced stages of culture (Figure 4). However, a notable disparity in conversion rates between the two methods was observed during the early cultivation phase at 48 h in this investigation: plate counting yielded a result of 34.2% ± 7.0, while flow cytometry showed only 3.3% ± 0.9. This discrepancy can be attributed to the presence of forespores at the early stage, where the forespore is encased by the plasma membrane and cortex, while the chromatin and bacterial structure of the mother cell remain intact (Errington, 2003; Higgins and Dworkin, 2012). Consequently, staining patterns of “spores that have not detached from the mother cells” under LDS751/SYTO24 dual staining align with vegetative cells rather than spores, resulting in the classification as Vegetative cells in flow cytometry analysis. These forespores exhibit heat resistance, thus are counted as spores in plate counting. Direct evidence of this inconsistency is provided by malachite green/safranin staining, which showed that at 48 h, distinguishable spores (blue-green) are visible within the bacterial body while most of the bacterial structure remains intact; by 60 h, bacterial cells degrade to an unobservable state (Figure 5A), with spores existing independently. Consequently, spore conversion rates determined by flow cytometry align closely with plate counting in later stages, suggesting that flow cytometry is more informative for calculating spore conversion rates during advanced *Bt* cultivation stages. Given that spores are typically harvested in the later fermentation stages in practical production settings, utilizing flow cytometry for spore conversion rate determination retains significant practical and guiding value.

The quantification of spore transformation rates through flow cytometry is influenced by factors such as cell and dye states, staining density, and machine consistency. It is crucial to control sample concentration, use separate dyes for each experiment, continuously monitor cell measurements, and standardize instrument settings to enhance inter-batch consistency across experiments. Despite stringent control measures, slight variations in fluorescence image alignment between batches may still occur. Therefore, increased monitoring of forward scatter (FSC) and side scatter (SSC) parameters within sub-populations can aid in accurately determining spore and vegetative cell cluster positions during fluorescence analysis. This approach improves the precision of flow cytometry in identifying cell sub-populations and assessing spore formation stages during cultivation. Such methodology offers a convenient and effective means of investigating the growth characteristics of different *Bacillus* strains, optimizing culture conditions, monitoring spore formation dynamically in large-scale *Bacillus* fermentations, and determining the endpoint of the fermentation process.

## Data Availability

The raw data supporting the conclusions of this article will be made available by the authors, without undue reservation.
