# Supplementary material for: Monitoring of Bacillus spore-forming dynamics through flow cytometry
Source: Front Microbiol. 2024 Oct 29;15:1450913. doi: 10.3389/fmicb.2024.1450913 (PMC11554475; doi:10.3389/fmicb.2024.1450913)
Supplement: Supplementary file 1 [file Data_Sheet_1.docx]

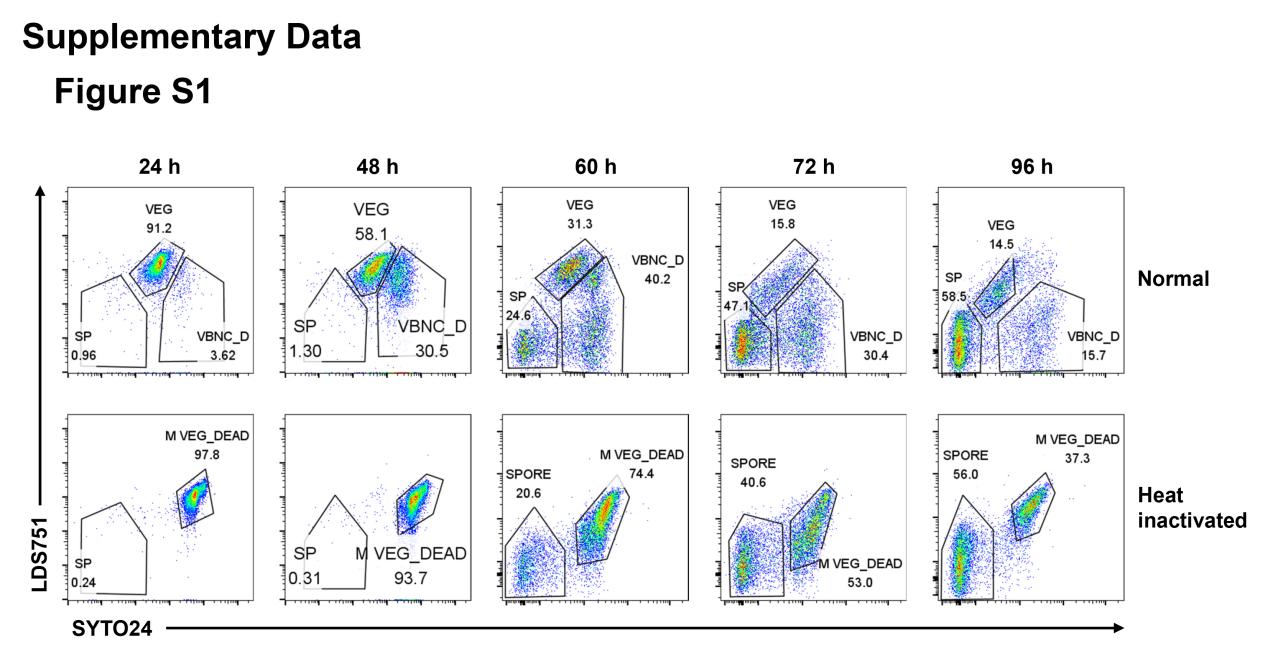


**Figure S1** Sub-populations of non-inactivated (normal, upper) and heat-inactivated (lower) *Bt* samples from different time points were analyzed by LDS751 and SYTO24.
